# The effect of working memory updating training on the Chinese writing ability of primary school students

**DOI:** 10.3389/fpsyg.2023.1163132

**Published:** 2023-05-23

**Authors:** Jiacheng Gao, Guangxin Li, Zimo Yang, Fengjuan Li, Tian Wang, Suxia Wen

**Affiliations:** ^1^Xinjiang Key Laboratory of Mental Development and Learning Science, College of Psychology, Xinjiang Normal University, Urumqi, China; ^2^College of Psychology, Xinjiang Normal University, Urumqi, China; ^3^College of Education Science, Xinjiang Normal University, Urumqi, China

**Keywords:** working memory, central executive, Chinese writing ability, updating, primary school

## Abstract

**Objective:**

This study aimed to explore the effects of working memory updating training on primary school students' writing ability and performance.

**Methods:**

A total of 46 fourth-grade Chinese primary school students were recruited; their performance in the Chinese character N-back training task, the Writing Ability Questionnaire, and a time-limited writing task was assessed.

**Results:**

The paired-sample *t*-test revealed that working memory updating training significantly improved the experimental group's working memory level. After training, a repeated measures ANOVA revealed that the experimental group's performance on the Writing Ability Questionnaire improved and was higher than that of the control group. In the time-limited writing task, independent-sample *t*-tests revealed that the experimental group's writing fluency increased and was higher than that of the control group, while the latter's grammatical accuracy and complexity decreased and were lower than those of the former.

**Conclusion:**

Working memory updating training can be used as auxiliary cognitive training to improve primary school students' working memory level, thereby promoting their writing development.

## 1. Introduction

Working memory is a memory system with limited capacity that provides individuals with a place to temporarily store information and is an important influencing factor in writing production (Baddeley, 1992). Working memory includes a phonological loop, a visuospatial sketchpad, a central executive, and episodic buffers. Among these functions, the central executive (consisting of updating, inhibition, and shifting) is the core component of working memory (Miyake et al., 2000). Working memory, particularly its updating, plays an important role in advanced cognitive processes (Zhao and Zhou, 2014).

Lower working memory levels are more likely to lead to writing problems (Baddeley, 2003; Thorell et al., 2009; Tagarelli et al., 2011; Wen, 2012; Archibald, 2017; Nielson and DeKeyser, 2019; Mavrou, 2020; Deldar et al., 2021; Vasylets and Marín, 2021; Li, 2022). Theoretical studies agree that there is intense competition for working memory resources during the writing process (Hayes, 2000; Kellogg et al., 2013). According to Skehan (1998), working memory resources have a competitive effect on the fluency, accuracy, and complexity of writing performance. Currently, a three-dimensional analytical architecture consisting of complexity, accuracy, and fluency is widely used in studies on the relationship between working memory and writing performance (Yi and Luo, 2013; Polio and Shea, 2014; Johnson, 2020). This is the writing performance construct used in this study.

The current research has focused on exploring the relationship between working memory and writing ability (Skehan, 2002; Vanderberg and Swanson, 2007; Kellogg et al., 2013; Capodieci et al., 2019; NoackLeSage et al., 2019; Sangani and Jangi, 2019; Sartori et al., 2021; Grace Kim, 2022). For example, studies have found that Chinese English as a Foreign Language learners' working memory is strongly related to fluency and syntactic complexity in writing performance (Yi and Ni, 2015a). Another study found that Chinese English as a Foreign Language learners' working memory has a significant effect on accuracy and syntactic complexity (Yi and Ni, 2015b). Supporting this view, one study found an independent effect of working memory on the complexity and accuracy of writing performance (Jin and Wang, 2021). Moreover, Vasylets and Marín (2021) found that the relationship between working memory and writing performance differed among learners with different writing proficiency levels. Working memory was associated with writing accuracy for low-writing proficiency learners, while working memory was associated with writing complexity for high-writing proficiency learners. Additionally, Zabihi (2018) found that higher working memory levels directly predicted higher writing complexity and fluency scores but negatively affected writing accuracy scores in a study of intermediate- and upper-level English learners. However, little attention has been paid to whether writing performance varies with changes in working memory levels. Several studies have found that working memory training can have positive transfer effects on reading comprehension and fluid intelligence in early childhood and school-aged individuals (Loosli et al., 2012; Peng et al., 2014), thus providing new ideas for further exploration of the causal relationship between working memory and writing ability. Gao (2019) verified that updating training can steadily improve Chinese writing performance among Chinese primary school students. However, aspects of individual writing performance that are enhanced by working memory training have not been studied. Examining the role of children's working memory transfer as an auxiliary in writing can enrich and enhance the current methods and efficiency of teaching writing to school-aged children.

The purpose of this study was to bridge a significant gap in the literature by comparing changes in individuals' writing ability and performance before and after working memory updating training. To solve this problem, we trained fourth-grade primary school students in working memory updating and recorded their writing test scores and performance.

Based on the limited extant research, we propose three hypotheses. First, working memory updating training can enhance primary school students' working memory performance. Second, the increase in participants' working memory proficiency can promote their writing ability. Third, the improvement of working memory level enhances primary school students' fluency-related writing performance.

## 2. Materials and methods

### 2.1. Participants

A total of 46 Chinese fourth-grade primary school students participated in this study. Participants were divided into an experimental group and a control group based on their scores on the Writing Ability Questionnaire (He, 2006). In total, four participants were excluded from the experimental group due to absence from school; thus, 19 participants (eight boys and 11 girls) remained. A total of five participants were excluded from the control group; thus, 18 participants (six boys and 17 girls) remained. The participants' ages at the beginning of the experiment were 9–11 years old *(M* = 10.02, *SD* = 0.49). The difference in mean age between the two groups of primary school students was not significant [*t*_(35)_ = 0.02, *p* = 0.99]. The students were from a class in the same neighborhood school, having similar language use experiences and living environments; the participants in both groups were right-handed and had no similar experimental experiences. None of the students participating in the experiment had psychiatric, neurological, or developmental disorders, according to previous assessments by the medical and mental health departments of the school. This study was approved by the Research Ethics Review Committee of the College of Psychology of Xinjiang Normal University, China. The students' parents were informed of the entire process, and consent was obtained from the students, guardians, and school before the experiment.

### 2.2. Research design

Between-participants design was adopted in the study. Both groups participated in normal school activities in the same class. Additionally, the experimental group underwent 14 sessions of working memory updating training over 8 weeks. The control group did not receive specialized working memory training. Participants in both groups were administered a writing ability test and a time-limited writing task test before and after training. The participants' writing ability tests were reviewed, data from the time-limited writing task were collected, and working memory updating training was conducted in a double-blind experimental setting.

### 2.3. Stimulus materials

#### 2.3.1. Updating training program

The experimental group performed an adapted Chinese-character N-back training task. Brain imaging studies have confirmed that the N-back task can sufficiently activate the brain regions associated with working memory and executive function (Richards et al., 2009). The training task was referenced to the N-back training task from previous studies (Zhao et al., 2013; Gao, 2019). The stimulus materials were referenced from the General Standardized Chinese Character List (State Council of the People's Republic of China, 2013), which was created by the Chinese Ministry of Education and the National Language and Character Work Committee, from which 500 Chinese characters were randomly selected as stimulus materials.

#### 2.3.2. Chinese writing tests and time-limited writing tasks

The Writing Ability Questionnaire was used to assess participants' writing ability. The questionnaire comprised four parts: reviewing, conceiving, expressing, and modifying abilities. The discrimination of each question was above 0.3, and the internal consistency coefficient (Cronbach's α) was *r* = 0.81.

The pre-test time-limited writing task was titled *My Vacation Trip*, and the post-test time-limited writing task was titled *A Day in My Life*. Both were paper-and-pencil tasks; the topics were related to the participants' daily lives and were of equal difficulty. None of these tasks had been performed by the participants in their usual studies.

The data recording method for the time-limited writing task was based on a previous study (Qi and Kim, 2022) in which the fluency, accuracy, and grammatical complexity of the participants' Chinese writing were recorded. As shown in Table 1, the term “fluency” refers to participants' writing speed, including the number of characters and words produced per unit of time. Accuracy and complexity introduced the concept of the revised T-unit to Chinese writing analysis (Qi and Liao, 2019). Consider a single sentence and a modified complex sentence as a T-unit. The clauses in the joint compound sentences were considered independent T-units. The term “accuracy” refers to the ratio of the number of error-free T-units to the total number of T-units in the participant's entire composition. The term “complexity” refers to the ratio of the average number of words contained in T-units, average number of words contained in sentences, and ratio of complex sentences to the total number of sentences in the composition. The two time-limited writing tasks were analyzed independently by two experimenters according to the study measures and verified by sampling with 93% sample consistency.

**Table 1:** Writing performance analysis indicators (Qi and Liao, 2019).

	**Indicators**	**Measurement methods**
Fluency	Number of characters written in a unit of time	Total number of characters divided by the time of writing
	Number of words written per unit of time	Total number of words divided by the time of writing
Accuracy	T-unit accuracy	Error-free T-units divided by the total number of T-units
Complexity	Average length of T-units	Total number of words divided by the total number of T-units
	Average length of sentences	Total number of words divided by the total number of sentences
	Proportion of compound sentences	Total number of compound sentences divided by the total number of sentences

### 2.4. Procedures

#### 2.4.1. Updating N-back training task

**Figure 1:**
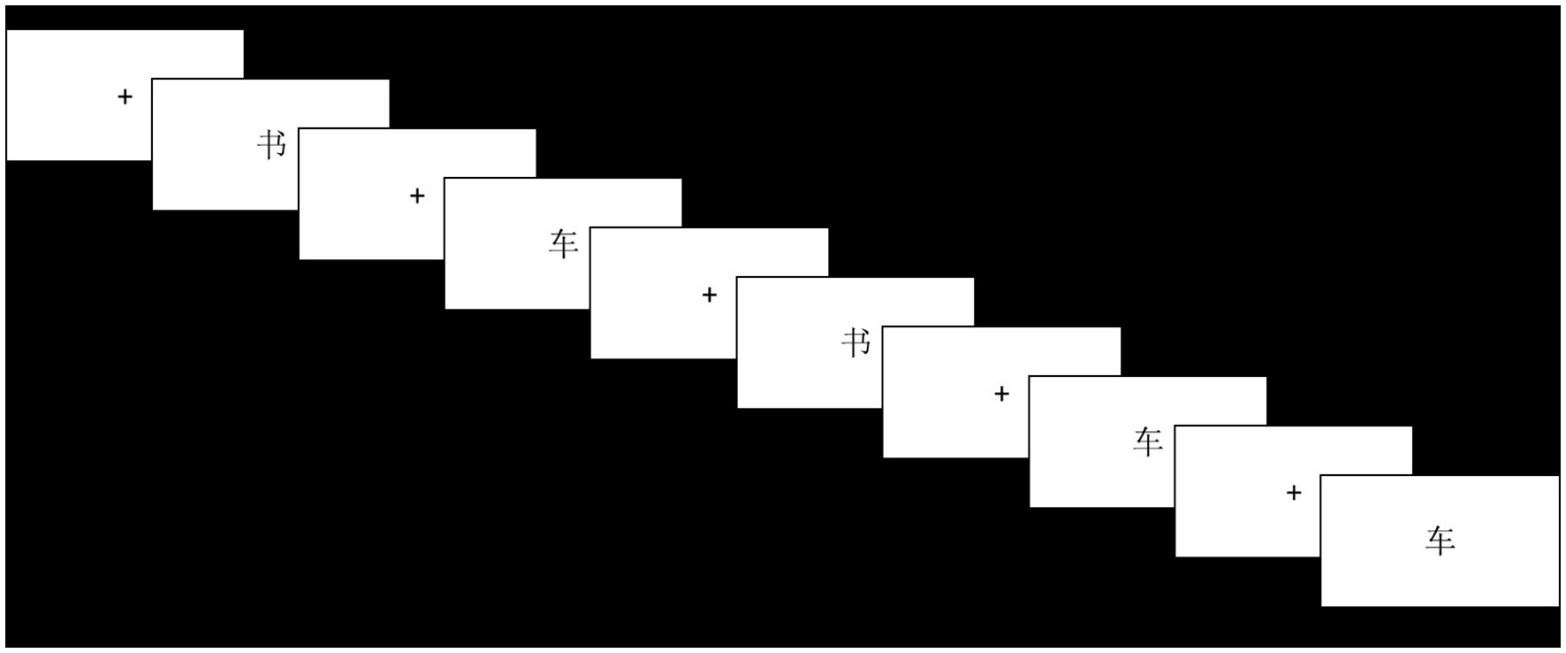
N-back training task.

The experimental group's training procedure was presented on a computer. There were four difficulties in the training task: 1-back, 2-back, 3-back, and 4-back tasks. Each difficulty level comprised 15 + n trials. As Figure 1 illustrates, each trial had a response time of 3,000 ms, and the participant needed to judge whether the current presented character was consistent with the previous nth-presented character and responded with a key press. In total, 10 trials were inconsistent, while five were consistent. All the trials were randomly presented. The participants were trained for 15 min each time, 1–2 times per week, for a total of 14 sessions. The training was conducted in a school computer room. The participants were familiar with the computer room, which was well-lit and ventilated, away from the classroom, and with low noise. Each participant underwent training daily in a standard classroom. In total, four monitors were present throughout the experiment, and they provided continuous feedback to the participants. The participants were given a sticker as a reward at the end of each training exercise. The first training session was conducted during the 4th week of the semester. At this time, the students' learning conditions stabilized. The participants in the experimental group started with 1-back training for each training session. At the end of each difficult task, the program provided participants with feedback on their accuracy during the task. If the accuracy rate was higher than 80%, the participants entered the next difficulty level. If the accuracy rate was lower than 80%, the participants were given one chance to repeat the difficult task. If the accuracy rate of the second training was below 80%, the difficulty level was decreased by one. Moreover, the 4-back tasks did not have an endpoint. When the total training time reached 15 min, the program automatically ended, and the accuracy and reaction time of the participants' training were recorded.

#### 2.4.2. Chinese writing tests and time-limited writing tasks

During the entire experimental stage, the two groups were administered a pre-training test and a post-training test, together with the Writing Ability Questionnaire and time-limited writing task. The pre-training test was conducted 1 week before the start of the working memory updating training. The post-training test was conducted 1 week after the completion of the updating training. The Writing Ability Questionnaire comprised 100 points and lasted for 60 min. The time-limited writing task collected only data on writing performance and lasted for 30 min. Both groups underwent the tests simultaneously.

We predicted that the performance level of the working memory updating training task would increase significantly in the experimental group after training. Meanwhile, participants in the experimental group exhibited higher writing ability test scores and improved writing performance compared with those in the control group. We used SPSS 20.0 software for all data analyses. Paired-samples *t*-tests of mean performance on the first 5 days of updating training and mean performance on the last 5 days of updating training for the experimental group were used to examine the changes in performance on the working memory updating training task for the experimental group. A 2 (subject type: experimental group vs. control group) × 2 (time: pre-test vs. post-test) repeated-measures ANOVA was used to examine the differences in the Chinese writing ability test scores between the two groups of subjects before and after training. An independent samples *t*-test was used to test the difference in the change in writing performance (post-training performance minus pre-training performance) between the two groups.

## 3. Results

### 3.1. Working memory updating training

**Figure 2:**
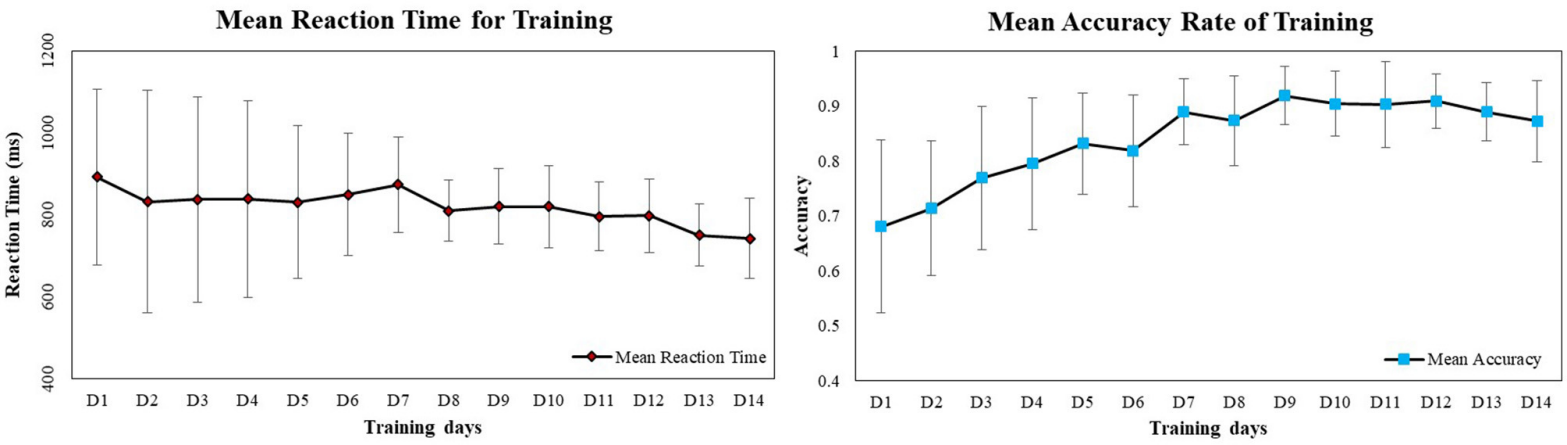
Mean reaction time and accuracy rate of training in the experimental group.

As shown in Figure 2, the paired-sample *t*-test of the mean scores of the first 5 days of updating training and the mean scores of the last 5 days of updating training in the experimental group revealed that working memory updating training enhanced the working memory performance (Hypothesis 1). The mean accuracy of the last 5 days of training was higher than that of the first 5 days of training [*t*_(18)_ = 6.24, *p* < 0.001, Cohen's *d* = 1.81, *r* = 0.67]. There was no significant difference in training reaction time [*t*_(18)_ = −0.75, *p* = 0.46, Cohen's *d* = 0.94, *r* = 0.42]. These results suggest that working memory updating training enhances working memory performance.

### 3.2. Chinese writing tests

A 2 (participant type: experimental vs. control group) × 2 (time: pre-test vs. post-test) repeated-measures ANOVA with both groups' Writing Ability Questionnaire scores revealed an increase in the experimental group's writing ability. As shows in Table 2, the interaction effect between participant type and time was significant [*F*_(1,35)_ = 12.20, *p* = 0.001, $\eta_{\text{p}}^{2}$ = 0.26]. A simple effects analysis revealed no significant difference between the pre-test scores of the two groups [*F*_(1,35)_ = 0.26, *p* = 0.61, $\eta_{\text{p}}^{2}$ = 0.01]. The experimental group scored higher than the control group on the post-test [*F*_(1,35)_ = 5.26, *p* = 0.03, $\eta_{\text{p}}^{2}$ = 0.13]. A simple effect analysis was conducted on both groups' pre- and post-test scores; the experimental group's post-test scores were higher than the pre-test scores [*F*_(1,35)_ = 267.34, *p* < 0.001, $\eta_{\text{p}}^{2}$ = 0.88]. The control group's post-test scores were higher than the pre-test scores [*F*_(1,35)_ = 121.87, *p* < 0.001, $\eta_{\text{p}}^{2}$ = 0.78]. The results indicate that teaching enhances writing ability and that an improved level of working memory can promote teaching effectiveness (Hypothesis 2).

**Table 2:** Mean pre- and post-training Writing Ability Questionnaire scores for both groups.

**Groups**	**Pre-training (*M* ±*SD*)**	**Post-training (*M ±SD*)**
Experimental group	33.84 ± 8.06	64.65 ± 12.48
Control group	35.11 ± 7.00	56.48 ± 8.74

### 3.3. Chinese writing performance

As shown in Table 3, independent sample *t*-tests for the change in writing performance (post-test performance minus pre-test performance) revealed a significant increase in writing fluency in the experimental group compared with the control group. The number of characters per unit of time increased and was higher in the experimental group than in the control group [*t*_(35)_ = 2.95, *p* = 0.006]. The number of words per unit of time increased and was higher in the experimental group than in the control group [*t*_(35)_ = 3.03, *p* = 0.005]. In terms of accuracy, the T-unit accuracy decreased and was lower in the control group than in the experimental group [*t*_(35)_ = 2.65, *p* = 0.01]. In terms of complexity, the average length of the T-units in the control group decreased and was lower than that in the experimental group [*t*_(35)_ = 4.78, *p* < 0.001]. The average sentence length in the control group decreased significantly compared with that in the experimental group [*t*_(35)_ = 2.88, *p* = 0.007]. The proportion of compound sentences decreased in both groups, but there was no significant difference between the groups [*t*_(35)_ = 1.22, *p* = 0.23]. The results indicate that the experimental group participants' writing fluency increased while maintaining their original accuracy and complexity. The control group exhibited increased writing fluency at the expense of accuracy and complexity (Hypothesis 3).

**Table 3:** Changes in writing fluency, accuracy, and complexity between the two groups.

		**Post-test minus pre-test**		***t***	**Cohen's *d***	***r***
		**Experimental group**	**Control group**			
Fluency	Number of characters written in a unit of time	5.54 ± 4.63	1.37 ± 3.92	2.95^**^	0.97	0.44
	Number of words written per unit of time	1.44 ± 1.43	0.11 ± 1.22	3.03^**^	1	0.45
Accuracy	T-unit accuracy	0.00 ± 0.13	−0.14 ± 0.20	2.65^*^	0.83	0.38
Complexity	Average length of T-unit	0.65 ± 1.28	−1.46 ± 1.40	4.78^**^	1.57	0.62
	Average length of sentences	−0.37 ± 4.09	−4.72 ± 5.06	2.88^**^	0.95	0.43
	Proportion of compound sentences	−0.07 ± 0.20	−0.14 ± 0.15	1.22	0.4	0.19

^*^p < 0.05. ^**^p < 0.01.

## 4. Discussion

This study examined the effects of working memory training on primary school students' writing using Chinese character N-back training, the Writing Ability Questionnaire, and a time-limited writing task. The results showed that working memory updating training improved primary school students' performance on the working memory task (H1) and the Writing Ability Questionnaire (H2). Additionally, the experimental group's writing performance showed a developmental pattern of increased fluency and maintained accuracy and complexity (H3).

The first and second findings indicate that working memory updating training and school teaching worked simultaneously for both groups of participants. Both groups scored significantly higher on the post-training writing test than on the pre-training test; however, in the post-training test, the experimental group scored significantly higher than the control group. This suggests that working memory updating training, as an auxiliary to normal teaching, can accelerate the development of individual writing skills. Our results are consistent with previous research suggesting that working memory training improves individual writing proficiency, but that working memory training cannot replace the role of teaching (Mo et al., 2018; Agha et al., 2022). The experimental results indicated that participants' writing ability improved through working memory training.

The third finding showed that the experimental group's writing fluency was significantly higher than that of the control group. This suggests that working memory training enhances individual writing fluency. These experimental results are similar to those of previous studies using working memory levels to predict writing performance in intermediate- and upper-level English learners (Zabihi, 2018). We filled the gap in the literature regarding the effect of working memory training on writing performance. Our experiment used working memory training to confirm the effect of working memory on writing performance. To the best of our knowledge, this is the first study to show that writing performance exhibits a developmental pattern of increased fluency while maintaining original accuracy and complexity through working memory training.

Writing is a complex process; it requires not only retrieving and transforming linguistic information from long-term memory into written form but also supervising the entire writing process and checking logical relationships in writing production. The entire writing process calls upon a large number of cognitive and linguistic resources (Li and Roshan, 2019). Working memory for temporary processing and storing information is important for supporting a successful writing process.

In elementary, low-level writing, learners pay more attention to word and syntactic processing, which are susceptible to working memory (Weigle, 2005). At this stage, each word occupies one unit of working memory. A high load on working memory for long periods leads to a decrease in the ability of the updating to continuously regulate and review working memory content during the writing process, which in turn leads to an increase in the error rate during the writing process. Learners with high levels of updating have significant learning advantages in these aspects of the writing process. They can allocate cognitive resources more effectively, balance the integration of meaning and form in the writing process, and perform multiple rounds of material analysis. They can even redistribute the remaining cognitive resources to help them reach advanced writing levels faster. In contrast, learners with low levels of updating have no choice but to engage in low levels of data-driven processing of writing information in situations where working memory is running under a high load and have no more resources to allocate to other parts of the writing process. On the one hand, this high load leads to an increased probability of mistakes in spelling and grammar, which are dominated by updating. On the other hand, it also reduces the degree of sentence-paragraph-chapter linkage in writing, thereby decreasing the overall quality of writing output.

This study confirmed that the improvement in participants' working memory performance through training promoted their writing performance. This finding supports previous research suggesting that working memory limits the process of writing output (Kellogg, 1996). This also supports the previous view that there is competition for cognitive resources, especially working memory resources, in the writing process (Skehan, 2002).

Updating's critical role in writing may be due to the fact that children first learn about writing systematically at the primary school level as well as their lack of proficiency in content during writing output. Children must repeatedly check the vocabulary and syntactic structures selected while writing. Furthermore, they must check what has been produced, as well as spelling, grammatical, and structural mistakes in writing. These writing output processes require a certain level of updating as a basis for ongoing conditioning and checking the content of newly entered working memory. An increased level of updating enhances the stability of the level of continuous regulation during individual writing and reduces the error rate under a high working memory load. It also promotes the performance of low-level learners and beginners in primary processing (e.g., wording). To some extent, it optimizes the monitoring system in the process of written language output, improves the efficiency of the reading and editing processes, and enhances the performance of the participants' language writing process. This conjecture was verified through our experiments.

As the teaching cycle progressed, the pattern of writing performance development in both groups revealed that improved working memory promoted increased fluency in the writing process and maintained previous levels of accuracy and complexity. In other words, individuals with high working memory proficiency produce more words and make fewer errors per unit of time in their writing. Therefore, based on the developmental differences between the two groups, we conjecture that both improved their writing fluency during normal educational activities. However, the experimental group's ability to monitor the writing process and maintain accuracy and complexity was enhanced, owing to the improved level of working memory. In contrast, the control group showed a significant decrease in accuracy and complexity because of further intensification of the working memory load while enhancing writing fluency, which increased the competition for working memory resources during the writing process.

Working memory updating training can enhance frontal middle gyrus activation, which is associated with writing (Westerberg and Klingberg, 2007). Thus, as the level of working memory increases, individuals gain access to more working memory resources to process and integrate writing processes. Individuals can maintain stability during high working memory loads while improving their capacity to monitor the writing process. This would compensate for the high error rate caused by students' weak connections to knowledge points and their unfamiliarity with the writing process at the primary level. This suggests that working memory updating training can be used as an auxiliary to writing education to accelerate the acquisition of individual writing skills.

Additionally, the retention of the effects of working memory updating training has been validated in previous studies. Peng et al. (2014) and Gao (2019) used working memory updating training to track the retention effects of working memory training for toddlers and primary school students, respectively, and found that working memory updating training continued to have a stable transfer effect on fluid intelligence and writing achievement 6 months after the end of training. This suggests that the transfer effect of working memory updating training is more pronounced in early childhood and school-age children. This view is indirectly supported by studies based on tracking primary school students showing that working memory is consistently a significant predictor of writing ability at the primary school stage (Guan et al., 2019; Rocha et al., 2022).

## 5. Limitations and prospects

Participants from the same group (class and community) were selected to balance their writing abilities, language experiences, and living environments. This similarity in teaching processes, writing strategies, and experiences may have led to similar developmental patterns in writing performance among individuals. These drawbacks may also explain the controversial results of current research on the relationship between working memory and writing. Additionally, the balance of participant proficiency allowed us to analyze only the differences in participants' writing performance and failed to provide in-depth statistical test results. Future studies should examine large sample sizes across various regions to balance the effects of participants' educational environment, writing strategies, and learning motivation. Alternatively, multiple small-sample studies should be conducted, strictly matching the factors of participants' educational environments, writing strategies, and learning motivation to examine working memory's effects on individual writing performance in different teaching modes. Ultimately, this will provide persuasive evidence for the numerous debates among researchers in this field.

## 6. Conclusion

This study confirms that an improvement in working memory can promote primary school students' Chinese writing performance. The participants showed a developmental pattern of writing performance in which fluency increased and previous accuracy and complexity were maintained. The three pieces of evidence we provide suggest that working memory training is an effective supplement for primary school students. Combined with the currently limited literature on the effects of working memory training on writing ability, these findings suggest that future research on working memory and writing ability should focus on the specific effectiveness of working memory training on writing, rather than simply assessing the relationship between working memory proficiency and writing ability. Working memory is one of the most important factors influencing the writing process, and future studies should provide a comprehensive understanding of writing development.

## Data availability statement

The raw data supporting the conclusions of this article will be made available by the authors, without undue reservation.

## Ethics statement

The studies involving human participants were reviewed and approved by the Research Ethics Review Committee of the College of Psychology, Xinjiang Normal University. Written informed consent to participate in this study was provided by the participants' legal guardian/next of kin.

## Author contributions

JG did the validation, data analysis, writing the original manuscript, writing-review and editing, and supervision. SW did the experimental design, supervision, and manuscript revision. GL did the experimental program design. ZY, TW, and FL did the data collection and calculation.
