# Supplementary material for: The effect of working memory updating training on the Chinese writing ability of primary school students
Source: Front Psychol. 2023 May 23;14:1163132. doi: 10.3389/fpsyg.2023.1163132 (PMC10242120; doi:10.3389/fpsyg.2023.1163132)
Supplement: Supplementary file 1 [file Table_1.docx]

Supplementary Material

**1 Writing Ability Questionnaire (Reproduced with permission from He, 2006)**

小学生写作能力测验（前测）

亲爱的同学：

您好！以下是一些同学在写作中遇到的问题和他们的想法，请您帮助他们选择正确的答案。另外，有些题目要你亲自做一做。

您在回答时，请把所选的答案序号写在括号（ ）里，记住：**题目答案并不是唯一的，可以选择多个答案**，只要您认为正确的都可以选。如果需要用文字回答的，则根据题目的要求把答案写在留出来的空白处。您的答案对我们的研究非常重要，您的所有答案我们都会保密，因此，请您尽己所能进行回答，不要有顾虑。

1、有一篇作文题目是“放学路上二三事”，你认为以下哪些想法正确： （ ）

①早上来学校时交通拥挤的情况。②中午放学路上看到的几件让人气愤的事。

③放学路上发生的几件有趣的事。④昨天语文课上发生的几件事

2、作文题目是“记假期里经历的一件事”，以下哪些内容写出来会好一些： （ ） ①和小伙伴一起玩电子游戏，玩得很开心。

②写假期中学做家务，受到大人们的表扬，很高兴也很自豪。

③写暑假姑姑从远方来，给自己买了很多礼物和自己非常喜爱的食物。

④写假期中练毛笔字，因为没练好，认识到做事情要有恒心有毅力，才能做好。

3、小明写作文“记我的一个好朋友”，他决定写好友小红，她很乐于助人。他想起了以下好几件事，请你帮小明同学选择合适的材料写这篇作文： （ ）

①小红在班上学习成绩很好。

②我生病请假，放学后小红来家中帮我补课。

③小红是一个爱清洁的人，经常洗头、洗澡、换衣服。

④小红找来一些材料帮助图书管理员 修补损坏的图书。

4、为写作文“放学以后”，一个同学准备了以下材料，请你帮他排出写作顺序： （ ）

① 比赛结果获得了第一名。

② 放学后赶去参加“小巧手杯”拼装模型比赛。

③ 最后 30 分钟抓紧时间组装。

④ 回家的路上明白了“功夫不负有心人”的道理。

⑤ 开始的时候非常细心，但速度有点慢。

5、以下是小红写的文章“台灯”，为了使人看懂，请你帮她排出写作顺序：（ ） ①它的底座是个黑色的玻璃盘，稳稳当当，显得庄重大方。

②一只红蓝相间的绢丝做成的灯罩，像一顶合适的帽子戴在灯泡上，美丽极了。

③在三个球之间，镶着银色铁圈，仿佛是两条紧身的腰带。

④我家有一盏约四十厘米高的台灯。

⑤往上，它分为三节。上下两节是六角型的玻璃球，乌亮鉴人，中间是一个乳白色的圆球，晶莹透明。

请你用下面的词造成一个句子，你还可以加入更多的内容，让句子写得既生动又具体。

6、 开 山上 月季花

|  |
| --- |
|  |

7、 天 大雨 下

|  |
| --- |
|  |

8、 天黑 怕

|  |
| --- |
|  |

9、以下是小明写的文章，请你帮她找出其中错误的地方并修改。

一天晚上，刚刚和强强在一起看电视小品节目。演员的精致表演，使他们二人不禁忍不住

|  |
| --- |

笑出声来。他俩边看边谈。他对他说:“言行不一致是一种极坏的作风。”强强听了，点头

|  |
| --- |

表示同意。

|  |
| --- |

**以下题目是了解你在写作过程中的情况：**

1、每题均有如下五个题项，请你选择最符合你写作实际情况的一项，并把相应的数字填在每个题目前的括号内。你的回答没有对错之分，请你凭第一印象快速地依次如实回答每个题目。

完全不符合 比较不符合 难以确定 比较符合 完全符合

1 2 3 4 5

（ ）1 我认真审题，知道要表达的主题是什么。

（ ）2 我围绕主题展开了联想。

（ ）3 我要采用一些新颖的素材，使自己的文章更具新意。

（ ）4 在着手写作之前，我要列出写作提纲。

（ ）5 我要思考文章那些地方需要详写，那些地方需要略写。

（ ）6 在写作文过程中，我要运用能够准确描述事物的词语。

（ ）7 我知道句子表达得是否正确，语法、用词是否有错误。

（ ）8 我知道我的文章有哪些需要修改的地方。

小学生写作能力测验（后测）

亲爱的同学：

您好！以下是一些同学在写作中遇到的问题和他们的想法，请您帮助他们选择正确的答案。另外，有些题目要你亲自做一做。

您在回答时，请把所选的答案序号写在括号（ ）里，记住：**题目答案并不是唯一的，可以选择多个答案**，只要您认为正确的都可以选。如果需要用文字回答的，则根据题目的要求把答案写在留出来的空白处。您的答案对我们的研究非常重要，您的所有答案我们都会保密，因此，请您尽己所能进行回答，不要有顾虑。

1、老师出的作文题目是“记我的一个好朋友”，你认为以下哪些想法正确： （ ）

①只要写自己的同学就行了。②决定写李刚，因为他是自己最要好的朋友。

③写自己的好朋友小华是个怎样的人，我喜欢他的原因。④认为自己有几个好朋友，决定每个都写。

2、老师布置作文“我爱看动画片”，请你从以下写作角度选择，从哪些角度写效果会好一些： （ ）

①动画片好看、有趣，又可受到教育。 ②写从动画片中能学到一些知识、开拓视野。

③写看动画片可以打发时间。 ④写小朋友爱看动画片，所以自己爱看动画片。

3、要写作文“星期天见闻”，为你准备了以下的材料，请你选出合适的材料：（ ） ①写星期天和妈妈上街，看到街上的人很多。

②写星期天和爸爸去看了一个有意思的儿童画展。

③写星期天去郊游所看到的美丽景色和农民伯伯的辛勤劳动。

④写星期天到表哥家玩，看到表哥家的小狗真好玩。

4、小红要写作文“养小兔”，她准备了以下一些材料，请你帮她排出写作顺序： （ ）

①学习养小兔的知识。 ②养小兔中的伤心事。 ③自己以为养小兔很简单，很容易。

④养小兔也要学习知识。⑤总结养小兔的体会。

5、一个同学要写文章“迟到”，他搜集了一些材料，请你帮他排出写作顺序： （ ）

①出门看见一个小妹妹在哭。

②帮小妹妹找到了妈妈。

③早晨一早就去上学了，怕迟到。

④从来不迟到的他，今天迟到了。

⑤小妹妹和妈妈走散了。

请你用下面的词造成一个句子，你还可以加入更多的内容，让句子写得既生动又具体。

6、 操场 同学们 跑

|  |
| --- |
|  |

7、 春天 美

|  |
| --- |
|  |

8、 妈妈 笑

|  |
| --- |
|  |

9、以下是小明写的文章，请你帮她找出其中错误的地方并修改。

②春天到了，赵老师带领我们到郊外活动。我们沿着一条平坦的公路，十分钟后，巍巍的山

|  |
| --- |

峰、辽阔的田野、曲折的小溪、别有情趣的乡间小镇，便展现在我们的眼前。看吧，美丽的

|  |
| --- |

小鸟在枝头快活地跳跃；五光十色的蝴蝶在花丛中翩翩起舞。大家都看着眼前的美景沉醉了，

|  |
| --- |

看着看着，我们不有自主地放漫了脚步。

|  |
| --- |

**以下题目是了解你在写作过程中的情况：**

1、每题均有如下五个题项，请你选择最符合你写作实际情况的一项，并把相应的数字填在每个题目前的括号内。你的回答没有对错之分，请你凭第一印象快速地依次如实回答每个题目。

完全不符合 比较不符合 难以确定 比较符合 完全符合

1 2 3 4 5

（ ）1 我认真审题，知道要表达的主题是什么。

（ ）2 我围绕主题展开了联想。

（ ）3 我要采用一些新颖的素材，使自己的文章更具新意。

（ ）4 在着手写作之前，我要列出写作提纲。

（ ）5 我要思考文章那些地方需要详写，那些地方需要略写。

（ ）6 在写作文过程中，我要运用能够准确描述事物的词语。

（ ）7 我知道句子表达得是否正确，语法、用词是否有错误。

（ ）8 我知道我的文章有哪些需要修改的地方。

**2 The Universal Standard Chinese Character List in N-back Training**

一 了 人 又 儿 十 二 力 几 入 八 九 七 厂 个 上 大 子 也 之 下 么 小 于 已 与 三 己 门 工 口 才 山 马 及 义 万 干 飞 千 广 习 土 不 中 为 以 心 天 见 方 无 手 开 分 日 文 从 什 长 公 气 太 月 少 今 内 计 水 王 化 书 比 风 五 认 元 车 反 办 区 切 火 引 六 片 队 支 专 历 斗 毛 他 们 出 可 去 对 生 用 只 发 头 本 主 正 外 打 处 民 白 由 业 四 加 平 立 边 叫 目 它 东 世 必 记 务 且 电 市 北 术 示 半 写 石 布 议 代 龙 包 节 号 史 在 有 她 地 那 会 过 自 好 而 后 多 如 行 成 年 此 当 同 老 回 动 问 全 因 向 关 先 再 机 名 并 合 西 次 安 件 光 色 则 至 设 任 各 式 许 场 产 交 论 传 决 军 华 百 红 江 收 达 众 存 共 约 观 农 导 权 列 级 团 争 价 划 米 压 阶 我 这 来 你 时 里 没 还 作 两 把 身 进 声 但 间 走 系 何 应 听 别 住 位 却 体 报 更 张 利 连 快 花 步 求 完 形 即 条 员 近 社 每 识 改 克 技 极 运 究 证 志 际 低 况 局 严 层 角 县 状 劳 的 到 和 国 事 知 所 法 经 其 定 现 实 学 明 话 些 者 使 表 性 果 放 制 直 非 命 变 建 受 图 该 往 或 转 物 金 取 空 拉 林 青 单 组 构 京 始 规 府 周 线 具 治 备 易 委 参 细 采 质 例 育 织 油 矿 是 说 要 看 面 前 点 将 种 相 信 很 便 重 给 结 统 亲 度 总 美 活 思 带 指 政 保 南 类 音 科 战 复 持 界 院 济 型 标 品 研 须 查 段 选 响 除 省 按 革 派 适 养 律 家 能 起 都 样 高 真 部 被 通 特 难 原 展 海 候 容 流 离 调 资 根 格 准 消 般 值 造 热 党 称 较 效 铁 素 速 料 验 积 圆 着 得 情 理 眼 做 接 常 据 第 教 象 清 基 深 领 断 商 维 率 族 道 就 然 等 最 程 提 斯 量 强 期 集 确 联 越 装 属 温 想 意 数 新 解 感 路 满 照 群 置 算 管 精 需 酸 题 影 增 器 整
